# Correlation Between Physiological and Performance-Based Metrics to Estimate Pilots' Cognitive Workload

**DOI:** 10.3389/fpsyg.2021.555446

**Published:** 2021-04-20

**Authors:** P. Archana Hebbar, Kausik Bhattacharya, Gowdham Prabhakar, Abhay A. Pashilkar, Pradipta Biswas

**Affiliations:** ^1^I3D Lab, Centre for Product Design and Manufacturing, Indian Institute of Science (IISc), Bengaluru, India; ^2^Council of Scientific & Industrial Research (CSIR)-National Aerospace Laboratories, Bengaluru, India

**Keywords:** flight simulator, EEG, ocular parameters, pupil dilation, saccades, cognitive load

## Abstract

This paper discusses the utilization of pilots' physiological indications such as electroencephalographic (EEG) signals, ocular parameters, and pilot performance-based quantitative metrics to estimate cognitive workload. The study aims to derive a non-invasive technique to estimate pilot's cognitive workload and study their correlation with standard physiological parameters. Initially, we conducted a set of user trials using well-established psychometric tests for evaluating the effectiveness of pupil and gaze-based ocular metrics for estimating cognitive workload at different levels of task difficulty and lighting conditions. Later, we conducted user trials with the NALSim flight simulator using a business class Learjet aircraft model. We analyzed participants' ocular parameters, power levels of different EEG frequency bands, and flight parameters for estimating variations in cognitive workload. Results indicate that introduction of secondary task increases pilot's cognitive workload significantly. The beta frequency band of EEG, nearest neighborhood index specifying distribution of gaze fixation, L1 Norm of power spectral density of pupil diameter, and the duty cycle metric indicated variations in cognitive workload.

## Introduction

It is well-known that pilot's cognitive workload has an impact on performance and, in turn, on flight safety. When workload is high, pilots pay less attention to the task at hand and their performance deteriorates due to narrowing of attention (Wanyan et al., 2011). Sufficiently low workload causes boredom, resulting in degradation in performance (Yerkes and Dodson, 1908). Designers need to consider these constraints for optimizing any pilot vehicle interface (PVI) designs. This is possible with an automatic estimation of pilot's cognitive workload. Other potential areas where pilot's cognitive workload estimation could be beneficial are:

- to design adaptive automation strategies based on human performance envelope (Thomas et al., 2015; Biella et al., 2017).
- to provide a basis for ergonomic design evaluation of aircraft cockpit display interface (Zongmin et al., 2014).
- to reason the causes of performance degradation for certain flight demands (Lee, 2010).
- to establish the performance limits for an aircraft with poor handling qualities (Harper and Cooper, 1986).
- to assist instructors in creating a sophisticated pilot assessment methodology (Ryffel et al., 2019; Rudi et al., 2020).

However, as flying an aircraft is a complex task, we need to consider innumerable physiological and psychological factors while evaluating pilot's cognitive workload. Even though subjective methods like NASA TLX questionnaire or Cooper Harper ratings are more prevalent in practice, researchers have proven that physiological variables are more sensitive for estimating cognitive workload (Causse et al., 2015; Trejo et al., 2015; Li et al., 2016). Several researchers have been exploring different physiological measures to quantify pilot's cognitive workload. Sharma et al. (2012) estimated pilot's cognitive workload using a spatial disorientation simulator and measured heart rate, respiratory rate, and galvanic skin responses. The study shows that physiological measures provide more valuable instantaneous information than subjective measures. Another finding of the study is that cardiac activity is a useful measure of cognitive processes. In another study, Othman and Romli (2015) employed multi-index evaluation to estimate cognitive workload, where the percentage of mean pupil dilation was evaluated along with subjective methods. According to a recent study by Mohanavelu et al. (2020), the effect of varying visibility conditions on pilots' cognitive demands could be evaluated through HRV features, pilot performance measures, and subjective assessment methods. It was found that even though pilots' performance scores were similar, the physiological measures were statistically significant. A comparative study by Gentili et al. (2014) revealed that when compared to EEG, HRV was less sensitive to variations in cognitive workload.

Hence, it is evident that researchers have extensively explored various psychophysiological measures such as brain-related measures (ERP, EEG, MEG, and brain metabolism), ocular measures (fixations, scan path, blinks, and pupil diameter), cardiac measures (HRV), and facial expression measures. However, there are few studies that correlate the different independent physiological and pilot performance-based parameters. There are even fewer studies that examine multiple measurement methods in a controlled experimental environment. This research work broadly covers the following objectives:

1. To ascertain the robustness of the proposed ocular parameters to distinguish variations in cognitive workload.
2. To design and conduct a realistic user experimental study using a flight simulator that simulates real-life flight environment as encountered by pilots.
3. To study various modalities of cognitive workload estimation and understand the significance of secondary tasks on pilot's cognitive workload.
4. To find relation among physiological measures such as eye gaze and EEG-based measurement and flying performance-based measures, and report differences among them.

We conducted two different user studies with the help of participants from our university. Ethical approval was taken from the Institute's Ethics committee for undertaking eye gaze tracking-based user studies. Written informed consent was also obtained from the participants for the publication of any potentially identifiable images and data used in this study. Our first study investigated differences in values of ocular metrics for standard psychometric tests in the laboratory to establish the robustness of the metrics to differentiate cognitive workload. In the second study, we conducted 36 flight simulator experiments with 12 participants. These trials were conducted for three different task scenarios. Participants' ocular parameters, EEG band power variations, and their flying performance parameters were recorded and analyzed. In total, we investigated 11 independent metrics to measure cognitive workload. This includes two pupil dilation-based ocular metrics, two gaze-based ocular metrics, variations in the median values of five different EEG frequency bands, and two pilot performance-based metrics. However, due to the limited resources available, other physiological measures such as heart rate variability and facial expression recognition could not be evaluated.

The choice of flight scenario was based on the recent incident and accident survey. A recent study by Boeing (2018)shows that taxing, climbing, approach, and landing are critical phases of civil aircraft flight. We have developed flight scenarios for taxing, take-off, and climb segments in the experimental flight simulator study reported in this paper. As the participants were non-pilots, scenarios were designed to increase task difficulty levels, starting from simple take-off, then with more monitoring and control requirements and additional secondary tasks. Results suggest that introducing the secondary task causes a significant increase in pilot's cognitive workload. This is observed in all the three estimated metrics, namely, EEG, ocular, and pilot performance-based metrics. Correlation between different parameters is explained in detail in section EEG Signal Analysis. Analysis results indicate a positive correlation among the three metrics.

This paper is organized as follows: The next section gives details of the literature survey of relevant research work in the field of cognitive workload estimation. Section User Study on Psychometric Tests presents the results of the comparison of ocular parameters for psychometric tests. Section Flight Simulator Study discusses the structure of the flight simulation experiments, followed by analysis of results. Section General Discussions deliberates on the results and the implications thereof. Section Conclusions concludes the results and discusses the future course of action.

## Related Research

There is a plethora of research articles that discuss cognitive workload measurement methodologies. In this study, we have dealt with three types of cognitive workload measures: EEG-based, ocular parameter-based, and flying performance-based metrics. Accordingly, our discussion in this section has been limited to the above methods only.

### Electroencephalogram (EEG) Signals

EEG is the measurement of brain's electrical activity. EEG signals are recorded through the EEG electrodes placed on the participant's scalp surface. Several studies validate that EEG power in different frequency bands is sensitive to changes in cognitive demand (Gevins et al., 1997; Petkar et al., 2009; Antonenko et al., 2010; Pavlov and Kotchoubey, 2017; Friedman et al., 2019). In a similar study, Cheng and Hsu (2011) estimated workers' fatigued state using EEG signal measurement. The study found out that an increased EEG activity in the theta band indicates decreased levels of attention. Borghini et al. (2012) introduced an EEG-based cerebral workload index to detect the driver's mental efforts during different levels of difficulty. This method was based on the estimation of increase in EEG power spectra. Schrauf et al. (2011) described EEG alpha spindles and alpha band power to be indicators of the driver's task performance during secondary auditory tasks. These findings in general suggest that EEG signal power levels are strong indicators of variations in cognitive workload.

### Ocular Parameters

Eye-tracking is a well-researched area of study for measuring cognitive workload (Hess, 1975; Kramer, 1991; Hyönä et al., 2003; Palinko et al., 2010; Babu et al., 2019). Ocular parameter-based measures for cognitive workload measurement can be categorized as pupil dynamics-based and fixation-based measures.

Studies suggest that the pupil dilates more with increase in cognitive workload (Marshall, 2007; Biswas and Langdon, 2015). Demberg and Sayeed (2016) study provides evidence of higher rates of rapid pupil dilations for more difficult task conditions. Prabhakar and Biswas (2018) study discussed evidence of using velocity of saccadic intrusion (SI) to detect the distraction of automobile drivers. The study also discussed the application of pupil dilation and fixation duration metrics for estimating cognitive workload. In a similar study, Abadi and Gowen (2004) used SI and micro-saccade rates to estimate cognitive workload. In another study, Xu et al. (2011) used non-intrusive remotely mounted eye trackers to measure variations of pupillary responses with cognitive workload. The study proved that pupil tracking is effective even with varying luminance conditions.

The distribution pattern of eye fixations is another proven cognitive load measure (Di Nocera et al., 2007). The visual scanning patterns in nominal environments tend to be deterministic and repetitive at regular intervals. The order of visual scanning tends to be more random with increase in cognitive workload. De Nocera suggested a widely used distance indicator called Nearest Neighbor Index (NNI) as a sensitive measure to perceive cognitive workload.

### Performance-Based Methods

Performance-based methods are indirect measures of cognitive workload. They are based on the assumption that an increase in task difficulty results in deterioration of performance, which increases the pilot's cognitive workload (or reduces the working memory capacity) (Wei et al., 2014). The simplest of these methods is the time domain statistical methods such as root mean squared error (RMSE), standard deviation of error, number of deviations outside tolerance, and computation of reaction time (Reising et al., 1995). For example, Smith and Caldwell (2004) conducted exhaustive simulated flight experiments to study pilot fatigue using RMSE. According to Ebbatson et al. (2007) how a pilot operates his/her control is also an indication of workload. Authors used power spectral density and autocorrelation coefficient of the control column data to infer pilots' control strategy.

Cognitive workload experienced by the participants is also indicated through his/her inceptor control strategy. Two such measures of pilots' efforts are the duty cycle (DC) and aggressiveness (Shepherd et al., 2009). Aggressiveness is the rate of change of inceptor control movements. DC indicates the percentage of time a participant controls his/her input on the inceptor. Hanson et al. (2014) have observed that an increase in aggressiveness and DC is an indicator of increased pilot workload.

To summarize, a variety of physiological and performance-based methods have been defined, tested, and validated to quantify pilot's cognitive workload. Correlation between different methods has also been reported in the literature. Bodala et al. (2016) inferred a positive correlation between pupil saccadic velocity and EEG theta frequency amplitude with increasing task difficulty. In another interesting study conducted by Scharinger et al. (2015) on investigation of working memory on reading comprehension, the authors concluded that pupil dilation dynamics functions as a global workload measure that includes motivational cognitive workload aspects. However, as per the authors, EEG band power is a more promising measure for identifying variations in cognitive processes. However, in another study by Borys et al. (2017), the authors reveal eye movement measures to be a good indicator of cognitive workload. The authors could not establish a significant relation between EEG and cognitive measures.

Hence, there have been such initial studies reporting the correlation between EEG and pupil dilation data analysis in basic research. However, to the best of the author's knowledge, there is limited published research carried out to investigate the relation between physiological parameters with flying performance-based parameters such as aggressiveness and DC. One of the aims of this study is to derive a correlation between the abovementioned parameters.

## User Study on Psychometric Tests

In this section, we describe a user study that was conducted to validate if L1 Norm of Spectrum (L1NS), Standard Deviation of Pupil (STDP), Low Pass Filter (LPF) of pupil diameter saccade rate, fixation rate, and median SI velocity can distinguish between different cognitive workloads of participants caused by task difficulty. Detailed description of metrics and their implementation can be found in Prabhakar et al. (2020). We used psychometric tests like the N-back test and arithmetic questions to assess the increase in participants' cognitive workload with increased task difficulty. We chose these tests as they were associated with working memory load (Marshall, 2007; Tokuda et al., 2011). Since the pupil dilation is sensitive (Beatty and Lucero-Wagoner, 2000; Vrzakova and Bednarik, 2012) to ambient light variation, we evaluated both the N-back test and arithmetic test in dark rooms as well as varying light conditions in the same room. While we evaluated the N-back test in both auditory and visual presentations, an arithmetic test was conducted only in auditory presentation.

We hypothesized that L1NS, STDP, LPF, saccade rate, fixation rate, and median SI velocity

1. are robust to ambient light variations
2. can be used to distinguish different levels of cognitive workload with respect to change in task difficulty of visual and auditory tasks.

### Participants

We collected data from 21 participants (16 male and 5 female) with an average age of 26 years from our university. We chose participants randomly such that the group had a mixture of people wearing and not wearing prescription lenses. Participants wearing lenses had either spherical or cylindrical or both types of powers.

### Materials

**Figure 1:**
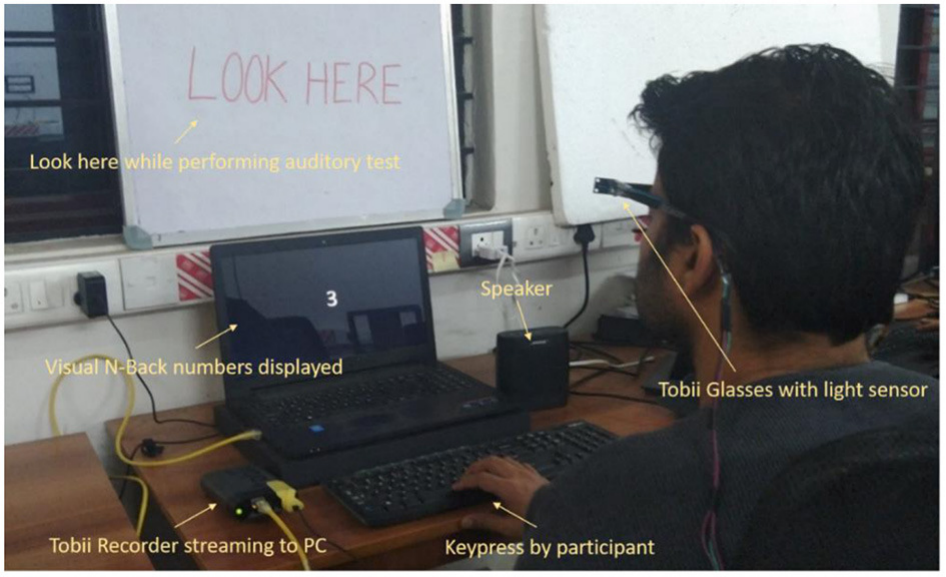
Participant performing the visual N-back test.

We collected data using Tobii Pro Glasses 2. We affixed two ambient light sensor modules, one sensor on either side of the glass frame, to capture illumination variations on both eyes independently (Figure 1). We used a Dell 17″ monitor to display numbers for visual N-Back and a Logitech keyboard to press the space bar to respond to the N-back test. We also used a Bose SoundLink speaker for an auditory cue in the auditory N-back test.

### Design

We undertook the following three tests:

1. Auditory N-back Test
2. Visual N-back Test
3. Auditory Arithmetic Test

The auditory tests were carried out in the dark as well as in dynamically varying light conditions. The room illuminance was varied from 0 to 150 lux by turning on and off a set of lights. The variation of illuminance was randomized.

#### N-Back Test

The N-back test had three levels of difficulties, viz., 1-back, 2-back, and 3-back. Participants were shown/spelled one stimulus (sequence of one-digit numbers from 1 to 9) in intervals of 2 s and had to press the space bar if the current stimulus matches the previous one (1-back), or second previous (2-back), or third previous (3-back). The N-back test levels were randomized to avoid the order effect. We developed software (Bjäreholt, 2014) to spell out/visually display numbers in N-back and to log the response from participants with a local time stamp.

#### Arithmetic Test

The arithmetic test had three levels, viz., easy, medium, and difficult. We developed a tool using python to read out questions using the Text-to-Speech engine in an arithmetic test. We recorded participants' response using the following steps:

1. Software read out all questions loudly.
2. Participant answered to questions loudly.
3. Instructor checked the answer and pressed the right/wrong key to log the event.

The difficulty levels were randomized to avoid the order effect.

### Procedure

Participants were asked to wear the Tobii glass affixed with light sensor modules. They were instructed to look at a poster pasted on the wall in front of them and to concentrate on the auditory task given to them. They were asked neither to close their eyes and nor to look around during answering the questions such that the tracker always detected eyes. Participants were explained about the N-back task and arithmetic task. They could practice the 1-back test before the actual trial in order to avoid the learning effect. The time stamps from logged events were used to synchronize the pupil/gaze data corresponding to the start and stop of N-back tests and arithmetic tests. We calculated L1NS, STDP, LPF, saccade rate, fixation rate, and median SI velocity corresponding to events. We checked if these metrics were high for 3-back compared to 2-back and to 1-back. We also checked if these metrics were high for difficult compared to medium and to easy arithmetic levels.

### Result

#### Performance of Tests

We measured performance of the tests as accuracy calculated from the confusion matrix as described in Table 1. The accuracy of the N-back test is calculated as

$$\begin{array}{l} \text{Accuracy}=\frac{correct+avoid}{correct+wrong+avoid+missed} \end{array}$$

and accuracy of the arithmetic test is calculated as

$$\begin{array}{l} \text{Accuracy}=\frac{correct}{correct+wrong} \end{array}$$

As the groups did not follow normality, we performed signed-rank test for each pair and found that accuracy of 3-Back/Difficult was significantly (*p* < 0.05) less than that of 1-Back/Easy for all the tests. The accuracy of 3-back/Difficult was significantly (*p* < 0.05) less than 2-Back/Medium for the auditory N-back dark room and both arithmetic tests. Accuracy of 2-back/Medium was significantly (*p* < 0.05) less than 1-back/Easy for visual N-back and auditory arithmetic dark room.

**Table 1:** Performance of the N-back test in terms of accuracy.

	**1-Back/Easy**	**2-Back/Medium**	**3-Back/Difficult**
Auditory N-back dark room	0.961 (0.073)	0.962 (0.059)	0.874 (0.122)
Auditory N-back dynamic light room	0.972 (0.084)	0.948 (0.090)	0.889 (0.121)
Visual N-back	0.985 (0.041)	0.942 (0.071)	0.891 (0.132)
Auditory Arithmetic dark room	0.992 (0.036)	0.905 (0.135)	0.770 (0.207)
Auditory Arithmetic dynamic light room	0.968 (0.067)	0.937 (0.134)	0.730 (0.318)

#### Visual N-Back (Pupil Dilation)

A repeated measure one-way ANOVA for metrics in Visual N-back is described in Table 2.

**Table 2:** Repeated measure one-way ANOVA for each metric with effect size.

L1NS Right eye	*F*_(2, 19)_ = 6.419, *p* < 0.05, η^2^ = 0.403
L1NS Left eye	*F*_(2, 19)_ = 33.964, *p* < 0.05, η^2^ = 0.781
STDP Right eye	*F*_(2, 19)_ = 7.849, *p* < 0.05, η^2^ = 0.452
STDP Left eye	*F*_(2, 19)_ = 29.408, *p* < 0.05, η^2^ = 0.756
LPF Left eye	*F*_(2, 19)_ = 30.718, *p* < 0.05, η^2^ = 0.764

**Figure 2:**
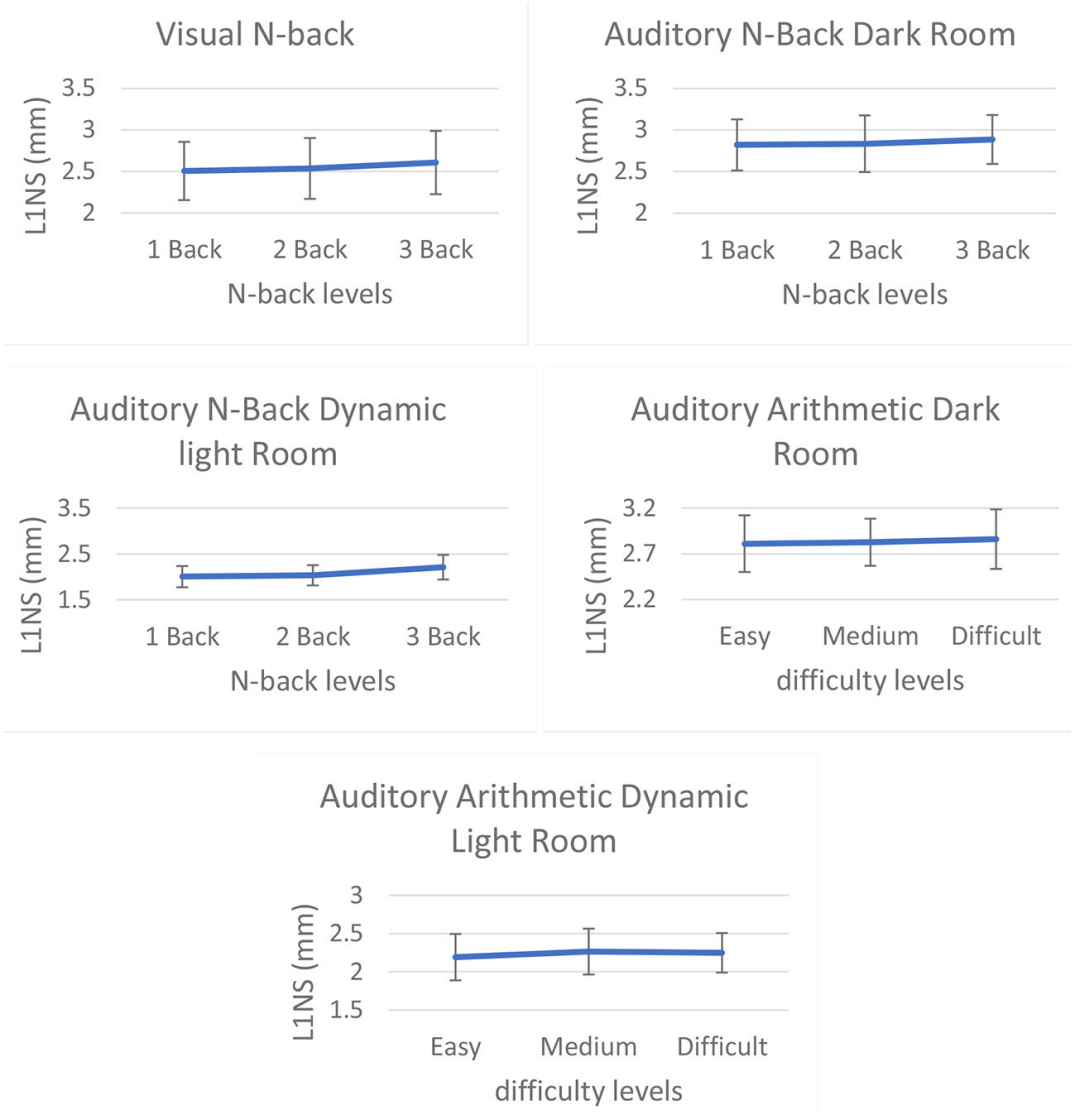
L1NS of the right eye for (from top left) visual N-back, auditory N-Back dark room, auditory N-back dynamic lightroom, auditory arithmetic dark room, and auditory arithmetic dynamic lightroom.

We found that L1NS and STDP of both eyes were significantly (*t*-test: *p* < 0.05) higher for 3-back than for 1-back. Similarly, 3-back was significantly (*t*-test: *p* < 0.05) higher than 2-back. We also found that LPF of the left eye was significantly (*t*-test: *p* < 0.05) higher for 3-back than for 1-back and higher for 3-back than for 2-back. We did not find any significant difference for saccade rate, fixation rate, and median SI velocity. A comparison graph of L1NS for visual N-back is given in Figure 2.

#### Auditory N-Back Dark Room (Pupil Dilation)

A repeated measure one-way ANOVA for metrics in Auditory N-back darkroom is described in Table 3.

**Table 3:** Repeated measure one-way ANOVA for each metric with an effect size.

L1NS Right eye	*F*_(2, 19)_ = 8.155, *p* < 0.05, η^2^ = 0.462
L1NS Left eye	*F*_(2, 19)_ = 7.813, *p* < 0.05, η^2^ = 0.451
STDP Right eye	*F*_(2, 19)_ = 5.91, *p* < 0.05, η^2^ = 0.384
STDP Left eye	*F*_(2, 19)_ = 15.842, *p* < 0.05, η^2^ = 0.625
LPF Left eye	*F*_(2, 19)_ = 18.088, *p* < 0.05, η^2^ = 0.656

We found that L1NS and STDP of both eyes, as well as LPF of the left eye, were significantly (*t*-test: *p* < 0.05) higher for 3-back than for 1-back. We did not find significant difference in saccade rate, fixation rate, and median SI velocity. A comparison graph of L1NS for auditory N-back in the darkroom is shown in Figure 2.

#### Auditory N-Back Dynamic Light Room (Pupil Dilation)

A repeated measure one-way ANOVA for metrics in Auditory N-back dynamic lightroom is described in Table 4.

**Table 4:** Repeated measure one-way ANOVA for each metric with an effect size.

L1NS Right eye	*F*_(2, 19)_ = 24.961, *p* < 0.05, η^2^ = 0.724
L1NS Left eye	*F*_(2, 19)_ = 43.017, *p* < 0.05, η^2^ = 0.819
STDP Right eye	*F*_(2, 19)_ = 29.461, *p* < 0.05, η^2^ = 0.756
STDP Left eye	*F*_(2, 19)_ = 39.767, *p* < 0.05, η^2^ = 0.807
LPF Left eye	*F*_(2, 19)_ = 38.847, *p* < 0.05, η^2^ = 0.804
LPF Right eye	*F*_(2, 19)_ = 28.797, *p* < 0.05, η^2^ = 0.752

We found that L1NS, STDP, and LPF of both eyes were significantly (*t*-test: *p* < 0.05) higher for 3-back than for 1-back. Similarly, 3-back was significantly (*t*-test: *p* < 0.05) higher than 2-back. We did not find a significant difference for saccade rate, fixation rate, and median SI velocity. A comparison graph of L1NS for auditory N-back in the dynamically lit room is shown in Figure 2.

#### Auditory Arithmetic Dark Room (Pupil Dilation)

A repeated measure one-way ANOVA for metrics in Arithmetic darkroom is described in Table 5.

**Table 5:** Repeated measure one-way ANOVA for each metric with effect size.

LPF Left eye	*F*_(2, 19)_ = 7.657, *p* < 0.05, η^2^ = 0.446
LPF Right eye	*F*_(2, 19)_ = 6.280, *p* < 0.05, η^2^ = 0.398

We found no significant differences for L1NS and STDP of both eyes. LPF of both eyes were significantly (*t*-test: *p* < 0.05) higher for 3-back than for 1-back. Similarly, 3-back was significantly (*t*-test: *p* < 0.05) higher than 2-back. We did not find a significant difference for saccade rate, fixation rate, and median SI velocity. We showed a comparison graph of L1NS for the auditory arithmetic test in the darkroom in Figure 2.

#### Auditory Arithmetic Dynamic Light Room (Pupil Dilation)

A repeated measure one-way ANOVA for metrics for Arithmetic test in dynamic lightroom is described in Table 6.

**Table 6:** Repeated measure one-way ANOVA for each metric with effect size.

L1NS Right eye	*F*_(2, 18)_ = 4.928, *p* < 0.05, η^2^ = 0.354
L1NS Left eye	*F*_(2, 19)_ = 5.966, *p* < 0.05, η^2^ = 0.386
STDP Right eye	*F*_(2, 18)_ = 4.790, *p* < 0.05, η^2^ = 0.347
STDP Left eye	*F*_(2, 19)_ = 4.595, *p* < 0.05, η^2^ = 0.326
LPF Left eye	*F*_(2, 18)_ = 7.662, *p* < 0.05, η^2^ = 0.460
LPF Right eye	*F*_(2, 18)_ = 6.648, *p* < 0.05, η^2^ = 0.425

We found that L1NS and STDP of both eyes were significantly (*t*-test: *p* < 0.05) higher for 2-back than for 1-back. We also found that the LPF of both eyes was significantly (*t*-test: *p* < 0.05) higher for 3-back than for 1-back. We did not find a significant difference for saccade rate, fixation rate, and median SI velocity. We showed a comparison graph of L1NS for the auditory arithmetic test in the dynamic lightroom in Figure 2.

#### Interaction Effect

We performed a repeated measure two-way ANOVA on metric values for factors like light, presentation, task type, and task difficulty and reported the metrics that showed a significant interaction effect between respective factors in Table 7 (tests of within-subjects effects) and Table 8 (multivariate tests). The factors and their levels are listed below.

1. Darkroom vs. dynamic lightroom (factors: light and task difficulty)
  - Darkroom (Auditory N-back) vs. dynamic lightroom (Auditory N-back)
  - Darkroom (Auditory Arithmetic) vs. dynamic lightroom (Auditory Arithmetic)
2. Auditory Arithmetic vs. Auditory N-back (factors: task type and task difficulty)
  - Auditory Arithmetic (Darkroom) vs. Auditory N-back (Darkroom)
  - Auditory Arithmetic (Dynamic lightroom) vs. Auditory N-back (Dynamic lightroom)
3. Auditory N-back vs. Visual N-back (factors: presentation and task difficulty)
  - Auditory N-back (Darkroom) vs. Visual N-back
  - Auditory N-back (Dynamic lightroom) vs. Visual N-back

**Table 7:** Tests of within-subjects effects.

**Interacting factors**	**Levels of factors**	**Metrics**	**Tests of within-subjects Effects (sphericity assumed)**
Light vs. Task Difficulty	Dark room (Auditory N-back) vs. dynamic light room (Auditory N-back)	STDP Left	*F*_(2, 40)_=19.369, *p* < 0.05, η^2^ = 0.492
		STDP Right	*F*_(2, 40)_=28.784, *p* < 0.05, η^2^ = 0.309
		L1NS Left	*F*_(2, 40)_=20.654, *p* < 0.05, η^2^ = 0.508
		L1NS Right	*F*_(2, 40)_=8.215, *p* < 0.05, η^2^ = 0.291
		LPF Left	*F*_(2, 40)_=18.881, *p* < 0.05, η^2^ = 0.486
		LPF Right	*F*_(2, 40)_=8.303, *p* < 0.05, η^2^ = 0.293
	Dark room (Auditory Arithmetic) vs. dynamic light room (Auditory Arithmetic)	STDP Left	*F*_(2, 40)_=3.708, *p* < 0.05, η^2^ = 0.156
		L1NS Left	*F*_(2, 40)_=3.394, *p* < 0.05, η^2^ = 0.145
Task Type vs. Task Difficulty	Auditory Arithmetic (Dynamic light room) vs. Auditory N-back (Dynamic light room)	STDP Left	*F*_(2, 40)_=18.229, *p* < 0.05, η^2^ = 0.477
		STDP Right	*F*_(2, 38)_=13.501, *p* < 0.05, η^2^ = 0.415
		L1NS Left	*F*_(2, 40)_=20.832, *p* < 0.05, η^2^ = 0.51
		L1NS Right	*F*_(2, 38)_=15.238, *p* < 0.05, η^2^ = 0.445
		LPF Left	*F*_(2, 38)_=17.889, *p* < 0.05, η^2^ = 0.485
		LPF Right	*F*_(2, 38)_=12.496, *p* < 0.05, η^2^ = 0.397
Presentation vs. Task Difficulty	Auditory N-back (Dark room) vs. Visual N-back	STDP Left	*F*_(2, 40)_=8.348, *p* < 0.05, η^2^ = 0.294
		L1NS Left	*F*_(2, 40)_=9.381, *p* < 0.05, η^2^ = 0.319
		LPF Left	*F*_(2, 40)_=8.855, *p* < 0.05, η^2^ = 0.307
	Auditory N-back (Dynamic light room) vs. Visual N-back	STDP Left	*F*_(2, 40)_=3.719, *p* < 0.05, η^2^ = 0.157
		STDP Right	*F*_(2, 40)_=4.979, *p* < 0.05, η^2^ = 0.199
		L1NS Left	*F*_(2, 40)_=3.444, *p* < 0.05, η^2^ = 0.147
		L1NS Right	*F*_(2, 40)_=4.807, *p* < 0.05, η^2^ = 0.194
		LPF Left	*F*_(2, 40)_=4.154, *p* < 0.05, η^2^ = 0.172

**Table 8:** Multivariate tests.

**Interacting factors**	**Levels of factors**	**Metrics**	**Multivariate test (Pillai's trace)**
Light vs. Task Difficulty	Dark room (Auditory N-back) vs. dynamic light room (Auditory N-back)	STDP Left	*F*_(2, 19)_ = 18.634, *p* < 0.05, η^2^ = 0.662
		STDP Right	*F*_(2, 19)_ = 8.398, *p* < 0.05, η^2^ = 0.469
		L1NS Left	*F*_(2, 19)_ = 20.084, *p* < 0.05, η^2^ = 0.679
		L1NS Right	*F*_(2, 19)_ = 7.375, *p* < 0.05, η^2^ = 0.437
		LPF Left	*F*_(2, 19)_ = 17.182, *p* < 0.05, η^2^ = 0.644
		LPF Right	*F*_(2, 19)_ = 6.747, *p* < 0.05, η^2^ = 0.415
	Dark room (Auditory Arithmetic) vs. dynamic light room (Auditory Arithmetic)	STDP Left	*F*_(2, 19)_ = 4.073, *p* < 0.05, η^2^ = 0.3
		L1NS Left	*F*_(2, 19)_ = 4.813, *p* < 0.05, η^2^ = 0.336
Task Type vs. Task Difficulty	Auditory Arithmetic (Dynamic light room) vs. Auditory N-back (Dynamic light room)	STDP Left	*F*_(2, 19)_ = 21.436, *p* < 0.05, η^2^ = 0.693
		STDP Right	*F*_(2, 18)_ = 11.517, *p* < 0.05, η^2^ = 0.561
		L1NS Left	*F*_(2, 19)_ = 20.509, *p* < 0.05, η^2^ = 0.683
		L1NS Right	*F*_(2, 18)_ = 12.273, *p* < 0.05, η^2^ = 0.577
		LPF Left	*F*_(2, 18)_ = 15.759, *p* < 0.05, η^2^ = 0.636
		LPF Right	*F*_(2, 18)_ = 10.168, *p* < 0.05, η^2^ = 0.53
Presentation vs. Task Difficulty	Auditory N-back (Dark room) vs. Visual N-back	STDP Left	*F*_(2, 19)_ = 10.415, *p* < 0.05, η^2^ = 0.523
		L1NS Left	*F*_(2, 19)_ = 11.691, *p* < 0.05, η^2^ = 0.552
		LPF Left	*F*_(2, 19)_ = 10.732, *p* < 0.05, η^2^ = 0.53
	Auditory N-back (Dynamic light room) vs. Visual N-back	STDP Left	*F*_(2, 19)_ = 3.717, *p* < 0.05, η^2^ = 0.281
		STDP Right	*F*_(2, 19)_ = 4.442, *p* < 0.05, η^2^ = 0.319
		L1NS Left	*F*_(2, 19)_ = 3.954, *p* < 0.05, η^2^ = 0.294
		L1NS Right	*F*_(2, 19)_ = 3.781, *p* < 0.05, η^2^ = 0.285
		LPF Left	*F*_(2, 19)_ = 3.872, *p* < 0.05, η^2^ = 0.29
		LPF Right	*F*_(2, 19)_ = 3.295, *p* < 0.05, η^2^ = 0.258

For Auditory N-back (Dynamic lightroom) vs. Visual N-back, LPF Right violated sphericity assumption, and we found a significant interaction between the factors using Greenhouse–Geisser as *F*_(1.453,29.059)_ = 5.094, *p* < 0.05, η^2^ = 0.203.

### Discussion

Our study confirmed the decrease in performance with increase in task difficulty (Granholm et al., 1996; Tokuda et al., 2011). We observed that L1NS, STDP, and LPF increased with increase in task difficulty, consistent with the study reported by Coulacoglou and Saklofske (2017). In all the cases, we observed that the parameter corresponding to a difficult task (3-Back and difficult arithmetic) was significantly higher than that corresponding to an easy task (1-Back and easy arithmetic). The intermediate task difficulty did not have a significant effect on all parameters. This might be because of the overlapping region of cognitive workload present in the 2-back test due to the transition of difficulty levels from 1-back to 3-back tests. Some participants would have found 2-back level as easy, and some would have found it difficult. Similarly, an overlapping region might be present in medium-level arithmetic questions. We found relatively large effect sizes in L1NS left eye for Visual N-Back, LPF left eye for Auditory N-Back Darkroom, L1NS left eye for Auditory N-Back Dynamic lightroom, LPF left eye for Auditory Arithmetic Dark room, and LPF left eye for Auditory Arithmetic Dynamic lightroom. Though STDP left and L1NS left were able to significantly distinguish between task difficulties in all conditions except dark room arithmetic, LPF left and LPF right were able to significantly distinguish between task difficulties in the darkroom arithmetic test. This infers that each metric performed significantly in each test condition. We also observed that the trend of increase in metric values with respect to increase in task difficulty is the same for changes in light conditions for visual and auditory presentations. Though we found an interaction effect between task difficulty and lighting conditions for pupil-based metrics, *t*-test results showed that our pupil-based metrics significantly distinguished between task difficulties in different lighting conditions. Similarly, a set of pupil-based metrics could substantially distinguish between task difficulties in different task types and presentation conditions despite significant interaction between the factors.

## Flight Simulator Study

Once the ocular parameters' robustness was evaluated with standard methods, we applied the same for aviation specific tasks. We conducted user studies in the high-fidelity NALSim flight simulator that is based on the Learjet aircraft model (Kamali et al., 2014). The purpose of the user study was to check the usability of EEG- and ocular parameter-based cognitive workload estimators and to investigate the effect of secondary tasks on cognitive workload.

**Figure 3:**
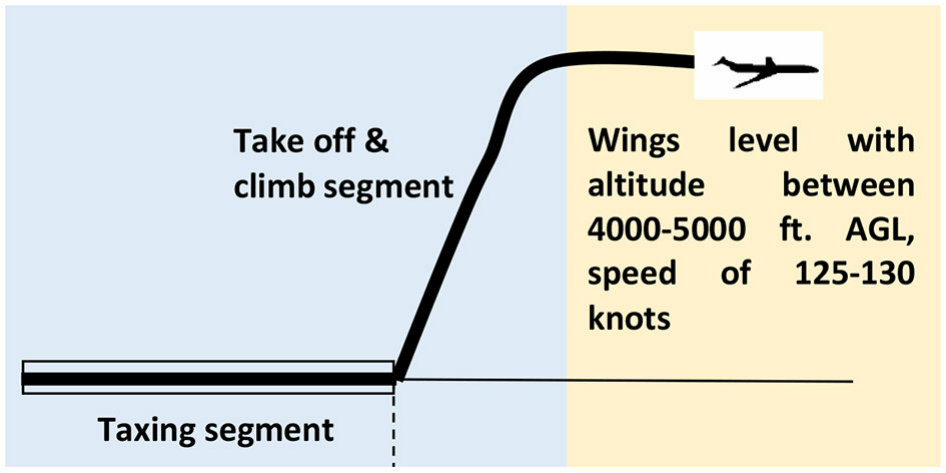
Test scenario.

All simulations started with similar initial conditions such as landing gear down, on ground, and a trim speed of 120 knots. The airport altitude was 89 0m above mean sea level. The baseline task was to conduct a controlled take-off, followed by a climb phase and a wings level flight (Figure 3).

### Participants

Biswas and JeevithaShree (2018) have suggested in their study that it is advisable to first test any new technology using participants with little or no knowledge about the platform. Accordingly, we chose students from our university for the flight simulator study. We conducted 36 simulations with 12 participants for three different task difficulty conditions C1, C2, and C3 mentioned in Table 9. Participants were aged between 22 and 40 years. The male-to-female ratio was 3:9. As the participants were non-pilots and new to the flight simulator environment, their cognitive workload variations were expected to be higher than that of experienced pilots (Antonenko et al., 2010). Hence, this sampling strategy satisfied our aim to relate different physiological parameters at varying levels of cognitive workload.

**Table 9:** Task scenarios for Flight simulator study.

**C1**	**C2**	**C3**
Take off: Initial checks, apply full throttle, take off at 130 knots speed. Climb and continue with a level flight for 4 min.	Similar to C1 till level segment. Maintain altitude between 4,000 and 5,000 ft above MSL for 4 min.	A secondary task was added in addition to C2 condition. The secondary task was defined as pointing and selection in an adjacent secondary head down touchscreen display.

### Design

We conducted 36 simulations with 12 participants for three different task difficulty conditions mentioned in Table 9.

The secondary task in C3 necessitated the participants to select a randomly positioned button in the secondary display based on an aural warning. While introducing the secondary task in C3, participants were instructed to prioritize their primary task, which was maintaining altitude within limits of ±1,000 ft.

### Materials

#### Simulation Setup

Simulation studies were conducted using the NALSim flight simulator at I3D lab, Indian Institute of Science, Bangalore, India. NALSim is a cost-effective ground-based variable stability flight simulator developed for Indian aircraft design programs. NALSim architecture is being used by a premier flight test pilot school of Indian Airforce for pilot training on aircraft handling qualities. The advantage of this simulator is that it is designed to provide a platform for researchers and aerospace students to understand aircraft dynamics, conduct aircraft configuration design studies, and handle quality studies and PVI studies.

**Figure 4:**
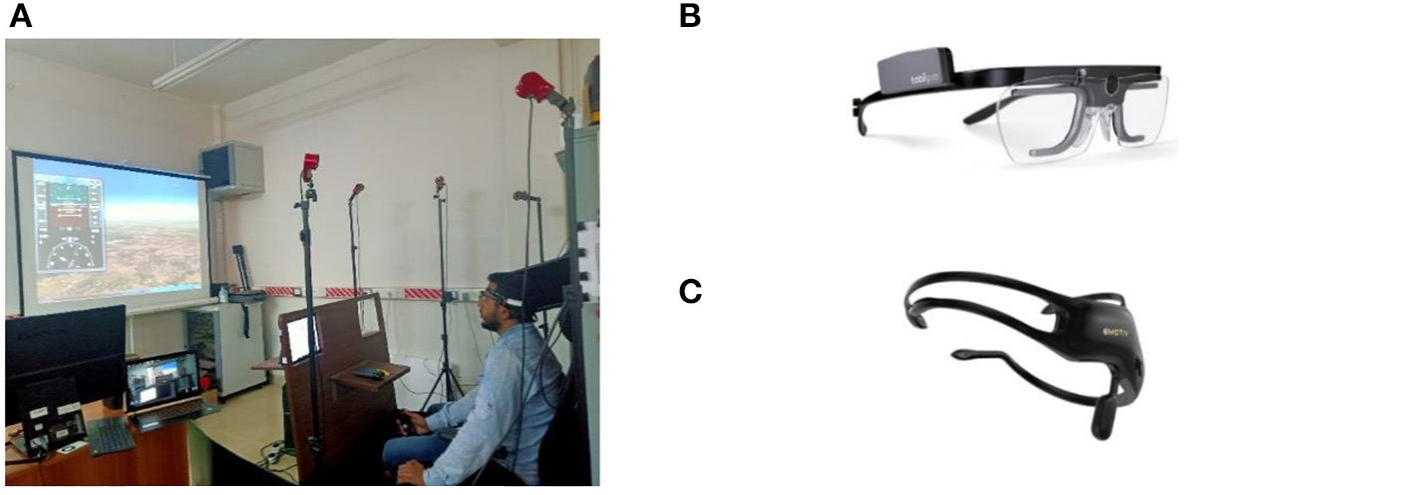
Apparatus. **(A)** Flight simulator setup. **(B)** Eye gaze tracker from Tobii [courtesy (14)]. **(C)** EEG headset from Emotiv [courtesy (15)].

The pilot's view in the simulator consists of out-of-the-window visual scenery and a head down display (Figure 4A).

#### Eye Gaze Tracker

Tobii pro wearable eye tracker (Figure 4B) was used in the study for recording eye gaze parameters (Eye tracking for research., 2018). This system measures three-dimensional gaze direction with an accuracy of 0.4° of visual angle. The sampling frequency of the device is 100 Hz.

#### EEG Headset

A study by Grummett et al. (2015) compares few inexpensive and wireless EEG systems for such experimental studies. The study supports the suitability of Emotiv headset for alpha responses and visual steady-state responses (VSSR). As the experiments designed in this study are related to VSSR, we have used portable, low-cost Emotiv Insight 5 channel EEG headset (Figure 4C) under controlled conditions for recording EEG data (Insight User Manual., 2018). EEG signals have a useful bandwidth in the range of the different frequency bands as given in Table 10.

**Table 10:** EEG signal bandwidths.

**Brain frequency bands**	**Frequency range (Hz)**	**State of mind**	**Changes in band power with increasing task demand**
Theta band	4–8	Sleepy, drowsy, meditative, and dreaming	Increases, associated with fatigue (Borghini et al., 2012) and information retrieval
Alpha band	8–13	Relaxed, calm, lucid state of mind	Increases with aural secondary task indicating increased information processing and reduced concentration ability (Schrauf et al., 2011)
Low beta (LB) band	13–21	Alert, active concentration, busy, and anxious state of mind	Increases (Pavlov and Kotchoubey, 2017)
High beta (HB) band	21–30	Focus, quick thinking, working	Increases (Pavlov and Kotchoubey, 2017)
Gamma	31–100	Optimal frequency for thinking, active thought, peak focus	Increases

The electrode positions that were studied are AF3 (left frontal), AF4 (right frontal), T7 (left temporal), T8 (right temporal), and Pz (central parietal).

### Procedure

We instructed participants to wear the EEG headset and the Tobii-pro eye-tracking glasses for the user trials. The contacts of the EEG headset were checked, and the eye tracker was calibrated prior to each simulation. All the participants were instructed about the procedure of the experiment and given 15 min of practice time to get acquainted with flying. It was ensured that participants were capable of performing wings level flight with constant altitude before starting the actual test scenario.

### Results

EEG, eye gaze, aircraft performance parameters, and inceptor control data were analyzed to infer the demand on pilot's cognitive workload. This section details the analysis results. We removed the outliers in the EEG and the gaze data using outer fencing. Normality in the data was checked using the Anderson–Darling test. As we found that both EEG and gaze data were not normally distributed, we used non-parametric tests such as Friedman test and Wilcoxon pairwise signed-rank test to analyze the significance of difference in cognitive workload.

#### EEG Signal Analysis

**Figure 5:**
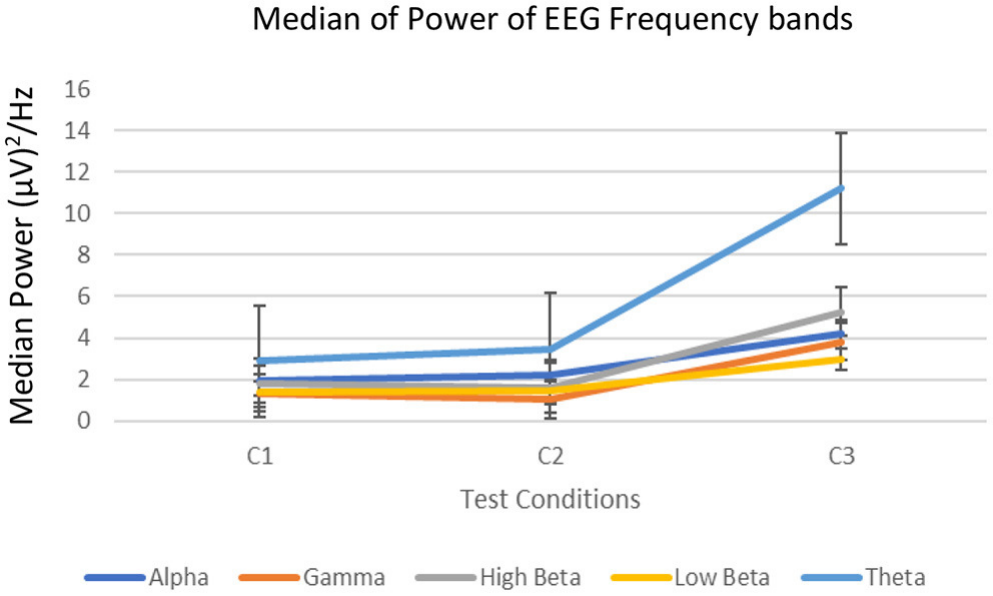
Median power of different EEG bands.

Figure 5 shows the median of power in each frequency band for C1, C2, and C3. We found that the median EEG signal power level increased from C1 through C3 in all the frequency bands. However, we did not find a significant increase in median power for the gamma frequency band (*p* > 0.5). Hence, we have not considered the gamma band for further discussions. The results from the statistical tests are summarized in Table 11.

**Table 11:** Statistical test results indicating changes in cognitive workload.

**Friedman Test**			
**Power levels in EEG Frequency bands**			
Alpha band			χ^2^(2) = 8.0, *p* < 0.05
LB band			χ^2^(2) = 8.166, *p* < 0.05
HB band			χ^2^(2) =10.5, *p* < 0.01
Theta band			χ^2^(2) = 10.66, *p* < 0.01
**Wilcoxon signed-rank test**			
	C1–C2	C1–C3	C2–C3
Alpha band	–	*Z* = 13, *p* < 0.05	*Z* = 16, *p* = 0.07
LB band	–	*Z* = 15, *p* < 0.05	*Z* = 13, *p* < 0.05
HB band	–	*Z* = 13, *p* < 0.05	*Z* = 10, *p* < 0.05
Theta band	–	*Z* = 1, *p* < 0.01	*Z* = 17, *p* = 0.07

We found that EEG signal power in the LB and theta band showed a significant increase between C2–C3 and C1–C3 (*p* < 0.05). Accordingly, C3 has a relatively higher cognitive workload.

#### Ocular Parameter Analysis

**Figure 6:**
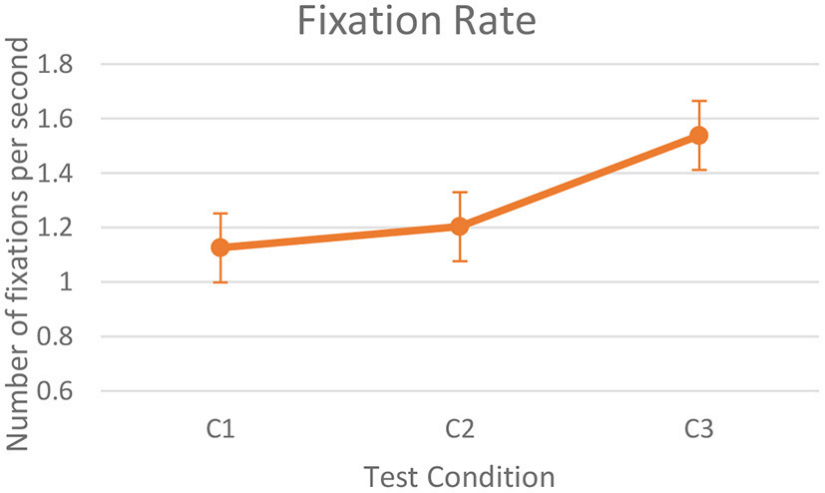
Fixation rate.

The following ocular parameters were selected for the flight 3 simulator study:

- **Gaze Fixation:** We used the fixation classification algorithm introduced by Tobii, called the Tobii I-VT filter (Olsen, 2012), for extracting fixations. According to Olsen, I-VT filter classifies eye movements based on the velocity of the eye's directional shifts. Gaze is classified as a saccade if the velocity is above a particular value of threshold (default−30°/s), otherwise, it is classified as a fixation.
  We computed fixation rate as the total time of fixations divided by simulation duration. Figure 6 shows the mean fixation rate for the three test scenarios. Friedman's statistics did not show any significant difference in the fixation rates between the test conditions. However, we observed an increasing trend of average fixation rate from C2 to C3 (*Z* = 1.88, *p* = 0.058).
- **Distribution patterns of fixation:** We used NNI in this study as an indirect cognitive workload estimator.

We computed NNI as the ratio of the nearest-neighbor distance (*d*_*NN*_) to the mean random distance (*d*_*MRD*_).

$$\begin{array}{l} NNI=\frac{d_{NN}}{d_{MRD}} \end{array}$$

Where $d_{NN}=\sum_{i=1}^{N}min[\frac{\sqrt{{\left(fx_{i}-fx_{j}\right)}^{2}-{\left(fy_{i}-fy_{j}\right)}^{2})}}{N}]\;$ and $d_{MRD}=\;0.5\sqrt{Area\;of\;interest/N}$

Area of interest is computed as the rectangular area that the *x* and *y* gaze coordinates cover. *fx* and *fy* are the *x* and *y* eye coordinates, respectively, and *i* and *j* are the successive time instances in *x* and *y*. *N* is the total number of data points.

**Figure 7:**
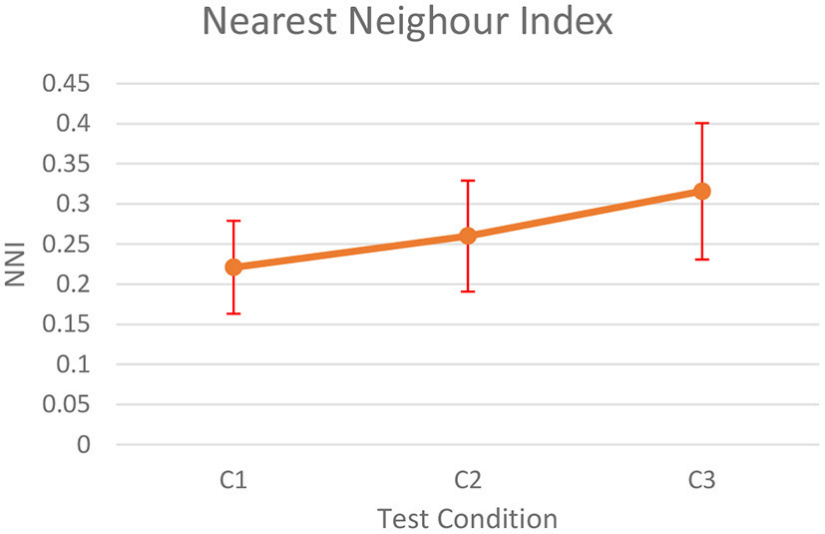
Nearest Neighborhood Index.

The mean of NNI scores for the three test conditions are shown in Figure 7.

We found that distribution of eye fixations significantly differs with different test conditions [χ^2^(2) = 9.50, *p* < 0.01]. Furthermore, pairwise comparison using signed-rank test showed that the eye fixations were more randomly distributed in space for C3 (*p* < 0.01 for C1–C3 and *p* < 0.1 for C2–C3).

c. **Pupil dilation dynamics:** We formulated the following metrics to extract features from the frequency spectrum of pupil dilation data. The three-frequency domain-based pupil dilation metrics discussed in section User Study on Psychometric Tests are L1NS, STDP, and LPF. In section Discussion, we found that L1NS shows the ability to significantly distinguish between task difficulties in all conditions. Hence, we used L1NS in the flight simulator study.

**Figure 8:**
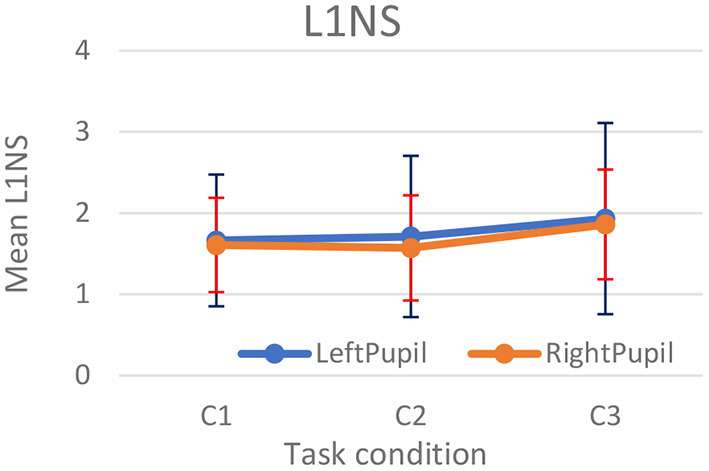
L1NS for left and right pupil diameters.

- L1 Norm of Spectrum (L1NS): Frequency domain-based L1NS on pupil dilation was computed based on the algorithm proposed by Prabhakar and Biswas (2018). Single-sided spectrum of the left and right pupil dilation time-series data (*Y*_*k*_) was computed using Fast Fourier transform given as:
  $$\begin{array}{l} \tilde{{\text{Y}}_{\text{j}}}=\;\overset{N}{\underset{k=1}{\sum }}Y_{k}{\text{e}}^{\left(\frac{-2\;\pi \text{i}}{\text{N}}\right)\left(\text{k}-1\right)\left(\text{j}-1\right)} \end{array}$$
  Frequency components from 1 to 5 Hz were summed to compute L1NS. Figure 8 shows the comparison plots for the left and right pupil diameters.
  We found that rate of change of pupil diameter was significantly different for right pupil for [χ^2^(2) = 6.17, *p* < 0.05, η^2^ = 0.2569]. Pairwise comparison showed that C3 had maximum changes in pupil diameters (*p* < 0.01 for C2–C3 and *p* < 0.1 for C1–C3).
- Median of SI velocity in °/second: As defined by Abadi and Gowen (2004), horizontal eye movements within 0.4° in the *X* axis where eye-gaze returns to the same position between 60 and 870 ms are known as SI (Prabhakar and Biswas, 2018). We used the algorithm described in Biswas and Langdon (2015) to compute median SI velocity.

**Figure 9:**
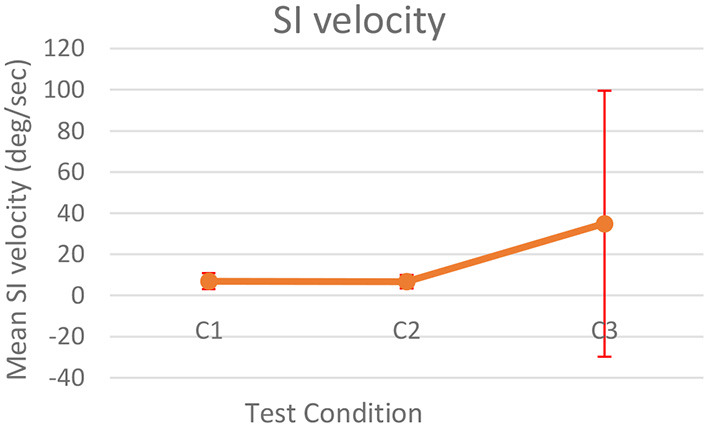
Median of SI velocity.

Figure 9 shows the median SI velocity for the three test conditions. Friedman test did not show any significant change in the rate of change of median SI velocity for the three conditions.

Table 12 consolidates the inferences of all the gaze measurement methodologies discussed in this section.

**Table 12:** Summary of analysis of ocular parameters.

**Friedman Test**			
Fixation rate			–
NNI			χ^2^(2) = 9.5, *p* < 0.01
L1NS			χ^2^(2) = 6.17, *p* < 0.05
SI			–
**Wilcoxon signed-rank test**			
	**C1–C2**	**C1–C3**	**C2–C3**
Fixation rate	–	–	*Z* = 1.88, *p* = 0.058
NNI	–	*Z* = 2.74, *p* < 0.01	*Z* = 1.65, *p* < 0.1
L1NS	–	*Z* = 1.8, *p* = 0.07	*Z* = 2.5, *p* < 0.01
SI	–	–	–

#### Flying Performance Analysis

We used DC and aggressiveness of participants' inceptor control columns as an indicator of cognitive workload experienced by the pilot. DC indicates the percentage of time a participant controls his/her input on the inceptor. DC is computed as follows:

$$\begin{array}{l} DC=100\%\text{*}\frac{1}{t_{n}-t_{2}}\overset{n}{\underset{i=2}{\sum }}x_{i} \end{array}$$

Here, $x_{i}=\left\{ \begin{array}{l} 0\;for\;\frac{\delta_{i}-\delta_{i-1}}{t_{i}-t_{i-1}}<noise\;threshold\;and\;\left|\delta_{i}\right|<\delta_{max}\\ \;1\;otherwise \end{array}\right.$

Aggressiveness is the rate of change of inceptor control movements. The formula is as follows:

$$\begin{array}{l} Aggressiveness=\sqrt{\frac{1}{n-1}\overset{n}{\underset{i=2}{\sum }}{\left(\frac{\delta_{i}-\delta_{i-1}}{t_{i}-t_{i-1}}\right)}^{2}} \end{array}$$

*t* is the simulation time, *n* is the number of data points, *δ*_*i*_ is the inceptor deflection in mm, and *δ*_*max*_ is the maximum stick deflection.

Plotting aggressiveness vs. DC is known as the PIW plot. Higher aggressiveness relates to more random control commands and higher DC infers that more time is required to control. We used the PIW plot to infer the variations in workload.

**Figure 10:**
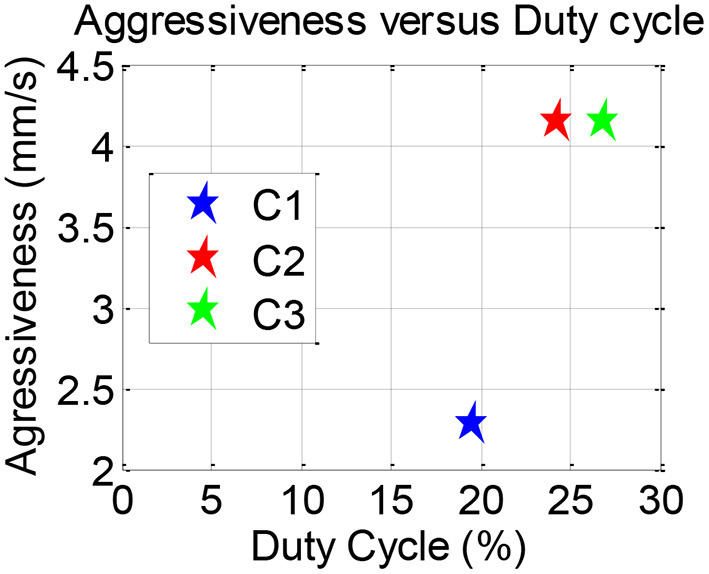
PIW plots.

Figure 10 shows the PIW plot of mean values of both the parameters for C1, C2, and C3. We observed that participants' aggressiveness levels were similar for both C2 and C3. However, participants had to spend more time controlling the inceptor in order to maintain level flight in case of C3 [*F*_(4, 26)_ = 2.72, *p* < 0.1, η^2^ = 0.247]. Furthermore, pairwise comparison using Tukey Kramer test showed that C3 had statistically higher DC than C2 (*p* < 0.1).

**Figure 11:**
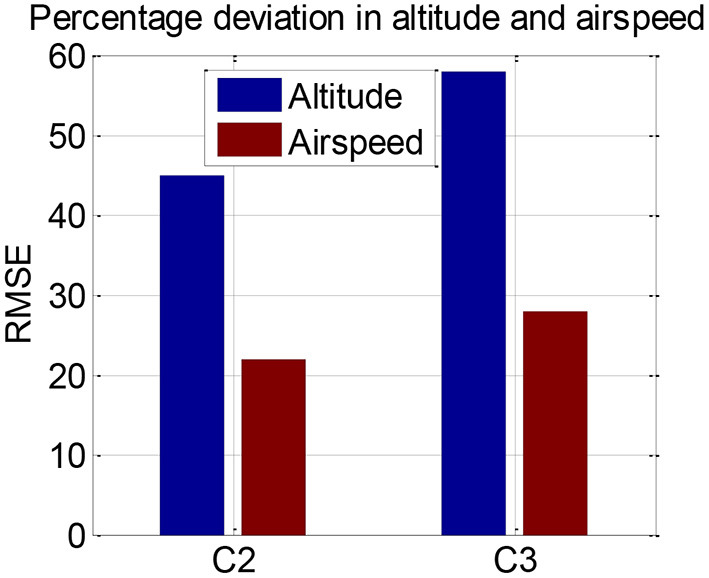
Percentage RMSE in altitude and airspeed.

Participants' inceptor control strategy, together with his/her flying performance, indicates his/her cognitive workload (Hebbar and Pashilkar, 2017). Accordingly, we computed flight performance in terms of RMSE in altitude and airspeed deviations (Figure 11). We found that errors increased significantly with additional demand of secondary task (for airspeed, *p* < 0.02).

### Conformance Among Parameters

Table 13 consolidates the results of cognitive workload estimation metrics discussed. A level of significance < 0.1 is indicated in green.

**Table 13:** Comparison between parameters.

	**Alpha band power**	**LB band power**	**HB band power**	**Theta band power**	**SI velocity**	**NNI**	**Fixation rate**	**L1NS**	**DC**
C1–C2	*p* > 0.1	*p* > 0.1	*p* > 0.1	*p* > 0.1	*p* > 0.1	*p* > 0.1	*p* > 0.1	*p* > 0.1	*p* > 0.1
C1–C3	*p* < 0.05	*p* = 0.05	*p* < 0.05	*p* < 0.01	*p* > 0.1	*p* < 0.01	*p* > 0.1	*p* = 0.07	*p* > 0.1
C2–C3	*p* = 0.07	*p* < 0.05	*p* < 0.05	*p* = 0.07	*p* > 0.1	*p* < 0.1	*p* = 0.058	*p* < 0.01	*p* < 0.05

In the case of C3, we found comparative similarity (*p* < 0.05) between LB and HB bands of EEG power, NNI and L1NS of ocular parameters, and the DC metric from flight parameters. Increased task difficulty was observed by the parameters mentioned above with secondary task (C3). However, ocular parameters such as SI velocity and fixation rate did not show a significant increase. This corroborates with the results from the psychometric study.

**Figure 12:**
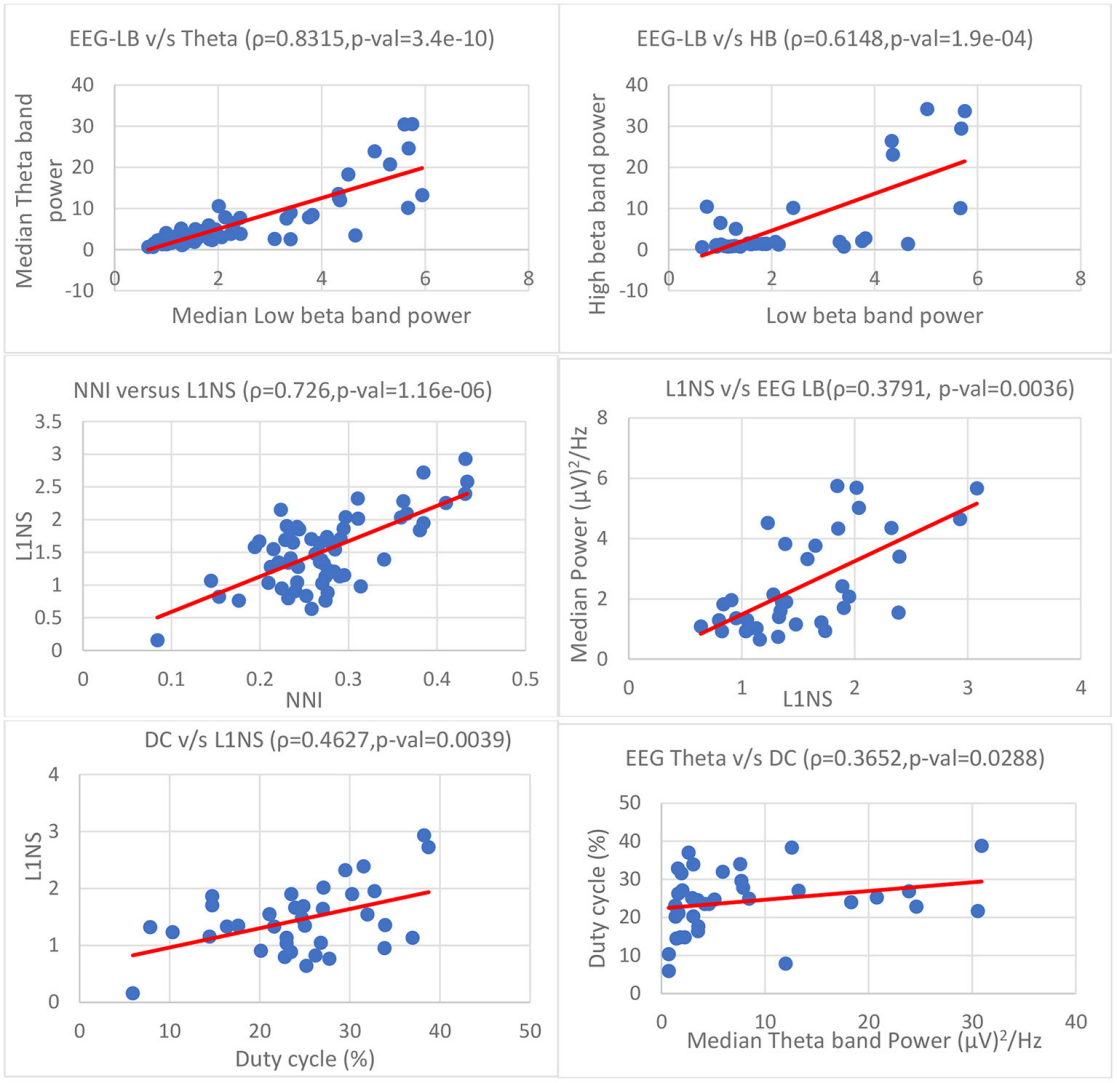
Correlation between EEG, ocular, and flying performance parameters (ρ is the pairwise linear correlation coefficient; p-val is the level of significance). (From top left) EEG: LB vs. theta, LB vs. HB; Ocular parameters: NNI vs. L1NS, L1NS vs. EEG LB; Flying performance: DC vs. L1NS, DC vs. theta.

Subsequently, we carried out Spearman's pairwise rank correlation analysis between the significant parameters given in Table 13 (Figure 12). Spearman's rank correlation coefficient (ρ) is computed as

$$\begin{array}{l} \rho =1-\frac{6\sum_{i}^{n}d_{i}}{n^{3}-n} \end{array}$$

Here, *d*_*i*_ is the difference between the ranks of each observation and *n* is the number of observations.

Firstly, we compared the correlation between the EEG parameters. We observed a consistent positive trend between LB, HB, alpha, and theta frequency band powers (0.6 < *ρ* < 0.9, *p* < 0.001). LB and theta bands showed a very strong association (ρ > 0.8, *p* < 0.001). When comparing the correlation between ocular parameters, we found statistically significant (ρ > 0.7, *p* < 0.001) positive correlation between NNI and L1NS.

Furthermore, we repeated the correlation analysis between EEG, ocular parameters, and the DC metric. Figure 12 shows the correlation plots for the significant parameters. We observed positive correlation between EEG and L1NS (LB and L1NS: ρ = 0.3791, *p* < 0.005; and theta and L1NS: ρ = 0.38, *p* < 0.005) and between EEG and NNI (LB and NNI: ρ = 0.4038, *p* < 0.05; theta and NNI: ρ = 0.4283, *p* < 0.05). We detected positive correlation between DC and EEG (DC and theta: ρ = 0.3652, *p* < 0.05 and DC and LB: ρ = 0.3338, *p* < 0.05), and DC and ocular parameters (DC and L1NS: ρ = 0.4627, *p* < 0.005; DC and NNI: ρ = 0.3251, *p* < 0.05).

### Discussions

Flight simulator studies were designed with three task conditions. The tasks were intended to increase the difficulty levels from C1 to C3. The recorded spectral power in beta and theta bands show a progressive increase from C1 to C3 and C2 to C3 (Table 14). These results have physiological significance in terms of human information processing. As discussed in Table 10, theta activity is associated with information retrieval. Beta band represents fast activity and is an indicative of increased thinking and focus levels. Hence, EEG data suggest that introducing a secondary task in C3 increased load on the participant's working memory. Results from ocular parameters also suggest a similar trend. NNI and L1NS showed a significant increase in C3. Hence, gaze fixations were more random and variations in pupil diameter were more predominant during C3. Data from the participant's flying performance and his/her control strategy [*F*_(4, 26)_ = 2.72, *p* < 0.0839, η^2^ = 0.247] also indicated increased cognitive workload with the inclusion of the secondary task.

Correlation analysis between EEG, ocular, and flying performance data for all the participants indicated positive correlation among all the parameters. Among the EEG frequency band power, LB and theta bands were found to be highly correlated. We also found that EEG theta and LB power, NNI, L1NS, and DC have a statistically significant positive correlation.

## General Discussions

We presented two studies on validating physiological measures to estimate pilot's cognitive workload in demanding scenarios. In the first study, different task difficulty levels were achieved through proven psychometric tasks such as N-Back and arithmetic tasks. As discussed in section Discussion, the test results confirmed our ocular parameters' robustness in estimating cognitive workload for varying task difficulties and varying illumination conditions. We found that L1NS, STDP, and LPF of pupil diameter were able to distinguish between different cognitive states corresponding to task difficulties irrespective of changes in lighting conditions. The results also proved that increase in task difficulty causes a decrease in performance. In our flight simulator study, manipulation of task difficulty was achieved in a controlled test environment with realistic flight scenarios. We used NALSim simulator and designed test scenarios that were representative of the real flight conditions. We used EEG, ocular, and flying performance parameters to estimate pilot's cognitive workload to address our third objective. We used standard statistical hypothesis methods to report the comparative results. Results from the user studies concluded that, in general,

- Distribution pattern of gaze fixations was more random with increase in task difficulty. This was proven by the NNI parameter in the flight simulator study.
- Pupil dilation-based L1NS metric showed significant increase in N-back and arithmetic tasks and aircraft flight task.
- In the case of EEG data, low beta and theta band powers were consistently more sensitive to task difficulty. Test condition with secondary task showed the highest cognitive workload among all scenarios.

We used participants' inceptor control strategy and their flying performance as another indicator for comparison with the physiological parameters. C3 showed higher DCs among the three test cases. The higher DC suggests that participants in the C3 test condition had to use the inceptor controls more rigorously than for other task conditions. Our final objective was to find a correlation between the multiple observations. We found that low beta and theta EEG band power, the gaze base ocular parameter NNI, the pupil dilation-based ocular parameter L1NS, and the performance-based parameter DC are indicators of cognitive workload variations and have positive correlation (*p* < 0.05) among themselves.

The primary aim of this study was to identify and correlate the different physiological and performance-based metrics as an indicative measure of pilot's cognitive workload. However, it is known that for the same task, novice pilots experience higher cognitive workload than experienced pilots (Antonenko et al., 2010). Hence, the results of the study are limited to understanding the correlation between the measures and not to compare the cognitive workload of the pilots. Future research would focus on validating the metrics discussed in this study with pilot evaluations for the entire flight envelope. Additionally, based on the available database, we plan to use machine learning techniques to classify pilots' cognitive status in real time.

These findings can also be extended to automotive domain where the drivers are always engaged in tasks that demand their attention and increase their cognitive load while driving. The estimated cognitive load from the proposed technique can provide necessary information to the car for making smart decisions when the driver undergoes increase in cognitive load.

Furthermore, cognitive workload estimation principles may turn out to be highly relevant for design optimization of any new product. Innovation starts with user's need, which is then fulfilled by creating new solutions or improving existing solutions. However, the challenge lies in identifying the real need of the users. Design thinking has been very successful in adopting a human-centered approach in identifying the need of the users in society. Hence, estimating a user's cognitive status is extremely critical to understand the underlying factors that govern responses of human mind and human actions. An accurate understanding of the cognitive processes can create an efficient design that can create a superior user experience.

## Conclusions

This paper discussed the application of non-invasive physiological measures along with task performance-based metrics to estimate pilot's cognitive workload. Initially, we conducted studies to estimate ocular parameters' ability to distinguish between variations in cognitive workload corresponding to differences in task difficulties. We also evaluated the robustness of our metrics in different ambient light conditions. In the second study, three different workload estimation methodologies were validated and compared. Participants were assigned different dimensions of task levels, such as primary and secondary tasks and maintaining one or many flight parameters. It was observed that the introduction of the secondary task (condition C3) along with flying caused a significant increase in cognitive workload. Degradation in performance due to such secondary tasks can be estimated from the proposed metrics. Thus, results discussed in this study propose a methodology for estimating pilot's cognitive workload based on his/her physiological measures such as EEG, ocular parameters, and the pilot's flying performance.

## Data Availability Statement

The raw data supporting the conclusions of this article will be made available by the authors, without undue reservation.

## Ethics Statement

Ethical review and approval was not required for the study on human participants in accordance with the local legislation and institutional requirements. The patients/participants provided their written informed consent to participate in this study. Written informed consent was also obtained from the participants for the publication of any potentially identifiable images and data used in this study.

## Author Contributions

PH has contributed in the formulation of the test cases, conducting the user studies, and analyzing the ocular parameters of the flight simulator experiments. KB has contributed in the conducting the user studies data extraction and analyzing the EEG data of the flight simulator experiments. GP contribution is the conduct of the psychometric test and analyzing the results. PB contribution is overall guidance, discussion of the results, and reasoning and conclusions. AP has provided overall guidance and support. All authors contributed to the article and approved the submitted version.

## Conflict of Interest

The authors declare that the research was conducted in the absence of any commercial or financial relationships that could be construed as a potential conflict of interest.
